# Emerging Trends in Biodegradable Microcarriers for Therapeutic Applications

**DOI:** 10.3390/polym15061487

**Published:** 2023-03-16

**Authors:** Harish K. Handral, Tom Adam Wyrobnik, Alan Tin-Lun Lam

**Affiliations:** 1 Stem Cell Bioprocessing, Bioprocessing Technology Institute, A*STAR, Singapore 138668, Singapore; 2 Department of Biochemical Engineering, University College London, Gower Street, London WC1E 6BT, UK

**Keywords:** microcarriers, biodegradable, mesenchymal stem cells, cell manufacturing, cell therapy, stem cells, regenerative medicine

## Abstract

Microcarriers (MCs) are adaptable therapeutic instruments that may be adjusted to specific therapeutic uses, making them an appealing alternative for regenerative medicine and drug delivery. MCs can be employed to expand therapeutic cells. MCs can be used as scaffolds for tissue engineering, as well as providing a 3D milieu that replicates the original extracellular matrix, facilitating cell proliferation and differentiation. Drugs, peptides, and other therapeutic compounds can be carried by MCs. The surface of the MCs can be altered, to improve medication loading and release, and to target specific tissues or cells. Allogeneic cell therapies in clinical trials require enormous volumes of stem cells, to assure adequate coverage for several recruitment locations, eliminate batch to batch variability, and reduce production costs. Commercially available microcarriers necessitate additional harvesting steps to extract cells and dissociation reagents, which reduces cell yield and quality. To circumvent such production challenges, biodegradable microcarriers have been developed. In this review, we have compiled key information relating to biodegradable MC platforms, for generating clinical-grade cells, that permit cell delivery at the target site without compromising quality or cell yields. Biodegradable MCs could also be employed as injectable scaffolds for defect filling, supplying biochemical signals for tissue repair and regeneration. Bioinks, coupled with biodegradable microcarriers with controlled rheological properties, might improve bioactive profiles, while also providing mechanical stability to 3D bioprinted tissue structures. Biodegradable materials used for microcarriers have the ability to solve in vitro disease modeling, and are advantageous to the biopharmaceutical drug industries, because they widen the spectrum of controllable biodegradation and may be employed in a variety of applications.

## 1. Introduction

Microcarriers are small particles, with sizes ranging between 50 and 400 µm, that have been extensively explored for cell manufacturing and used as drug carriers [1]. Microcarriers with customizable design, materials, and size have been of huge interest in the biomedical field for broader application in tissue engineering, 3D bioprinting, and in vitro disease modeling platforms [2,3,4]. Attempts have been made to fabricate microcarriers with different biomaterials such as cellulose, chitosan, collagen, dextran, gelatin, biopolymers, and many others [5,6], see Table 1. The biomaterial’s properties are primarily responsible for the physical features, such as size, geometry, topography, stiffness, and porosity, of microcarriers [7]. Various microcarrier fabrication methods have been implemented to achieve the desired, controllable physical attributes, among which, emulsification and microfluidics-based methods are the most promising [8].

Microcarriers are becoming a more popular method for scaling up cells towards cell therapy applications. As cell treatments become more widely used in clinics, more streamlined scale-up, process robustness, cost efficiency, and regulatory compliance are required. Obtaining several billion cells per batch is a requirement at the large scale, however, it is laborious to achieve such cell quantities using traditional tissue culture flasks [9]. Monolayer cultures have been shown to lead to loss of ECM proteins, and abnormal cell morphology and phenotype [10]. During the harvesting and expansion, additional modifications at genetic and epigenetic levels can occur [11,12]. Three-dimensional (3D) cultures are expected to provide, and better mimic, the native physiological tissue architecture, thus retaining the cells’ morphological and functional attributes. Manufacturing of cells via microcarriers and bioreactor platforms has shown promising results, without hampering the cells’ healthy state [13,14]. Additionally, liquid–liquid phase separation (LLPS) is a new method for creating microcarriers for biological purposes. The phase separation of LLPS microcarriers in an aqueous solution, results in the production of discrete liquid phases, with differing compositions. This enables the encapsulation of various bioactive compounds, such as proteins and growth factors, in various phases of the microcarrier [15,16]. Cell harvesting from substrates is challenging in conventional microcarrier systems, because it requires enzymatic treatment, which is frequently paired with agitation. Authors of a recent study investigated a two-phase system for hMSC expansion and non-enzymatic cell harvesting. Perfluorocarbon droplets were disseminated in a protein-rich growth medium, and employed as temporary liquid microcarriers for hMSC culture [17].

LLPS microcarriers have shown promise in drug delivery, tissue engineering, and cell therapy applications. Furthermore, these microcarriers are biocompatible, biodegradable, and adjustable in terms of their physical and chemical properties [18].

Extensive research has been reported on the use of biocompatible and biodegradable materials in numerous applications including drug delivery [19,20,21], tissue engineering [22,23,24,25], cell manufacturing, and bioprinting [26,27,28,29]. In such applications, biodegradable microcarriers, combined with bioreactors, play a key role in meeting the demand for cell expansion. For example, traditional methods of cell seeding and harvesting from microcarriers require the use of dissociation chemicals or enzymes to separate cells, potentially affecting cell yield and risking a greater apoptotic cell population. Studies highlighting the aspects of biodegradable microcarriers that are advantageous for enhancing their therapeutic value to cells, are presented in Table 1; and a quantified data set of different types of coating materials used on microcarriers, and their impact towards cell growth, expansion, and nutrition perfusion is presented in Table 2.

Overall, many promising reports on the design and optimization of microcarriers that are adaptable for xeno-free, scalable, and implantable systems, with the capacity to modulate cell responses, show that their use is an ever-growing trend in the biomedical and cell therapy space.

## 2. Key Features of Microcarriers for Therapeutic Applications

Mesenchymal stem cells (MSCs) are among the most extensively investigated cell-based therapeutic products that have reported significant applications in tissue repair, immune modulation, and regeneration [30,31]. The ability of manufacturing platforms to enable the growth of living cells for a broad patient pool, as well as to achieve a robust, efficient, and scalable process, to fulfill commercial demand, is a major challenge [32,33,34,35]. Scale-up manufacturing platforms that include the use of microcarriers, could be tailored for cell-specific expansion and formulation, to enhance the vital functional properties of cells. Here, the fabrication of microcarriers should consider cell-specific requirements, to achieve high cell yield and a lower population of apoptotic cells, so that ultimately the clinical effectiveness of the cells is enhanced [36,37]. The most critical feature of microcarriers is the ratio of surface area to volume, offering the growth of large populations of cells in a relatively small culture vessel, consuming less growth medium [38]. The matrix materials used for microcarrier fabrication are critical for cell growth and harvesting. For example, surface coatings such as polylysine, poly(N-vinylguanidine), and poly(N-isopropylacrylamide) (PNIPAAm), on microcarriers, could facilitate MSC cell attachment, bead-to-bead transfer of cells, nutrient perfusion, as well as promoting differentiation into a variety of mature cells of interest [39,40]. The spectrum of stiff to soft substrates has also enhanced the properties of cells to differentiate, and affected marker expression, the cell secretome, and immunomodulatory features. Cells respond to a Young’s modulus ranging from 10 to 1000 kPa, depending on whether they differentiate to neural, fat, cartilage, or bone. Surface-coated microcarriers with ECM proteins (collagen, fibronectin, and vitronectin), or derivative motifs, can enhance the cytoskeletal organization, and change cellular morphology, activate intercellular signaling pathways, or control gene expression [41,42,43]. During cell amplification, an appropriate biomimetic microenvironment could therefore support cell proliferation and help retain biological functions. Along with mechanical stability and stiffness profiles, biophysical cues such as porosity (between 60% to 90%), hydrophilicity, or nanopatterns (e.g., 10–50 µm star-shaped design), could promote cell yields, by modulating cell behavior and differentiation abilities [44].

**Figure 1:**
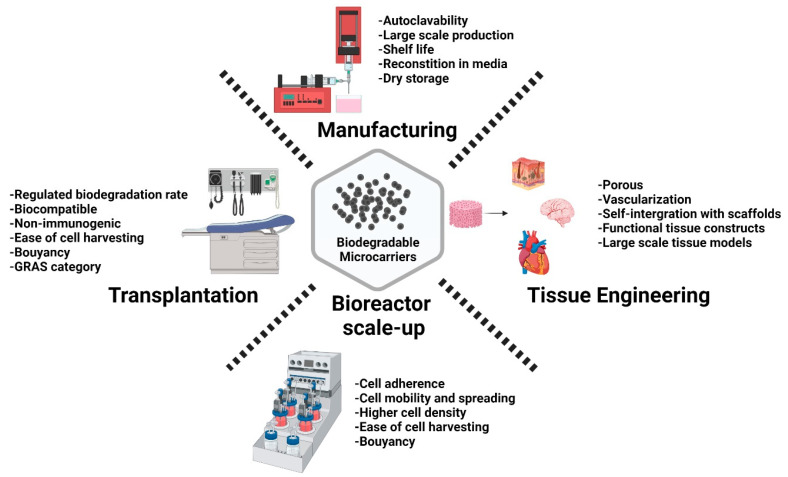
Schematic illustration highlighting the considerable features of biodegradable microcarriers, for their applications in cell manufacturing and regenerative medicine. Created with Biorender.com (accessed on 28 September 2021).

Along with scale-up considerations, attempts have been made to understand cell biology, such as the secretome, fate upon clinical infusion, integration with tissues, proangiogenic properties, and crosstalk with immune cells, and what soluble factors could amplify the clinical effectiveness, as depicted in Figure 1. The intended biological properties of the cell should provide guidance for process development, by understanding how to optimally design and fabricate microcarriers or scaffolding support structures for the respective cell types. Shedding light on biodegradable materials would aid in choosing the appropriate materials for tissue regeneration [45].

## 3. Biodegradable Microcarriers for Cell Manufacturing

Due to the limited amount of adult stem cells that can be retrieved from patients, it is necessary to generate large amounts of stem cells outside the human body, with a cost-effective approach. The use of microcarriers is an established technology in the biopharmaceutical industry, which, in combination with stirred-tank bioreactors, can provide the necessary environment for large-scale production of adherent cells. However, conventional microcarriers have been regarded as a potential safety risk to the patient, because particulates may remain in the final product. As such, traditional microcarriers have not been classified as cGMP compliant, which has hindered their widespread use in clinical trials or production processes for previously authorized autologous stem cell products [31]. As a result, adult stem cells such as MSCs, even in clinical settings, are often still cultured in poorly controlled and labor-intensive two-dimensional tissue flasks. The development of microcarriers that can be dissolved in vitro, or degraded in vivo, could represent a major step forward in overcoming the existing challenges in stem cell expansion, and open opportunities for the use of volumetrically scalable bioreactors [32,46]. In the case of dissolvable microcarriers, the cells could be harvested without the use of the traditional enzymatic dissociation method, by pH, temperature, biochemical changes in adherent molecules, changes in protein chemistry of surface receptors, and other biochemical changes that do not hamper the cells’ adherence features. Depending on the speed of degradation, cells growing on biodegradable microcarriers could be harvested by dissolving the microcarriers within the bioreactor, or both cells and microcarriers could directly be implanted into the site of injury [47]. A recent report, showcased the use of porous PLGA microcarriers for the culturing of human adipose stem cells, which remained undifferentiated in dynamic culture conditions [34]. Microcarriers were evaluated for stability at 37 °C, to cultivate cells, and found to be stable with no signs of degradation for up to two months in water, at 4 °C. The biodegradability and other bioengineering confirmation studies were reported in Muoio et al., which demonstrated the gradual degradability of the microcarriers under stirred conditions at 37 °C, when cultured for up to nine days [33].

Likewise, for large-scale expansion of therapeutic cells, dispersible and dissolvable porous microcarrier material (3D TableTrix^TM^) has been developed, and identified for use in stirred bioreactors [35]. Briefly, 3D TableTrix^TM^ has been designed with a dispersible and dissolvable feature, that aids in avoiding the need for time-consuming microcarrier separation from cells, and its soluble property offers a higher rate of cell recovery. Authors have reported the potential use of this application in cell manufacturing, by showing 500-fold multiplication of adipose-derived mesenchymal stem cells (AdMSCs) in a 1 L bioreactor system, with a final cell yield of 1.05  ±  0.11  ×  10^9^ hMSCs, with 98.6% recovery rate in 11 days, cultured under serum-free conditions. Furthermore, cells maintained their differentiation abilities to trilineage, stable genomic profiles, as well as immunophenotypic profile, while exhibiting negligible signs of senescence.

Cultispher G, a cross-linked porous microcarrier, is commonly employed as a cell carrier in cell therapy applications. In stirred tank bioreactor culture, such gelatin-based microcarriers support a wide range of adherent cell types, and are scalable to hundreds of liters. Cultispher G is particularly beneficial, since it can be enzymatically dissolved, making cell harvesting easier, without the need for cell-bead filtering [36]. The delayed destruction of deposited ECM, by enzymatic reagents, on the other hand, inhibits the cell recovery rate, decreasing cellular viability. The invention of the stimuli-triggered breakdown of cross-linked microcarriers for cell harvesting, has addressed these issues. In comparison to conventional beads, newly produced redox-sensitive beads (RS beads) have exhibited faster disintegration, allowing for greater hMSC dissociation, with significant cell yield after culturing for eight days [37]. The concept has been tested and demonstrated in spinner flasks, as well as bioreactors. In comparison to Cultispher G beads, studies were conducted to ensure that surface modification of the microcarriers (RS beads) did not affect cell adhesion. After cell adhesion and growth in spinner flasks, the redox dissolving time for RS beads was found to be faster than the enzymatic dissociation time for conventional beads. MSCs grown on the RS beads did not show any significant difference in the growth curve, compared to the control regular beads. Interestingly, the cell harvest time in 3 L bioreactors, for cells cultured on RS beads, was at least 15 times more rapid than the control [37]. RS beads show great potential as cell carriers in manufacturing applications, as they allow for cell proliferation with higher recovery yield.

Porous microcarriers are commonly used to grow, expand, and harvest stem cells. In most cases, the cells are harvested using proteolytic enzymes, which can result in cell damage. One of the studies developed a variety of alginate/PEG (AL/PEG) semi-interpenetrating network of microcarriers, to overcome such limitations. The interaction between the carboxylic acid group of alginate and the di-terminated amine groups of cystamine, was applied, to chemically cross-link alginate and PEG, to form networks. PEG was added to regulate the degradation of the microcarriers, and actively interact with the alginate network. Furthermore, the mechanical stability of the AL/PEG complex, was enhanced by the electrostatic characteristics of chitosan coated on the surface. Non-coated AL/PEG microcarriers exhibit poor mechanical stability, and this is worse when non-cross-linked PEG molecules are discharged into the culture medium. A chitosan coating was used to boost the mechanical stability of AL/PEG, and, as the authors expected, AL/PEG microcarriers with the chitosan coating had a greater cell proliferation rate, and after 5–7 days of culture, a 12-fold increase in cell yield was observed [38]. The results revealed that PEG size and molecular weight modulated the microcarriers’ properties. Furthermore, the microcarriers were engineered to degrade when disulfide links were cleaved. The rate of microcarrier degradation was tuned, depending on changes in the AL to PEG ratio, the amount of chitosan coating, and the type and concentration of reductant utilized. AL/PEG microcarriers have also been developed to aid in the attachment and proliferation of MSCs. Therefore, a reductant overcame the constraints of cell harvesting from microcarriers, while also decreasing the cell damage induced by proteolytic enzyme treatment, and enhanced the cell yield.

Yan et al. (2020) demonstrated the use of porous microcarriers for the culturing of adipose-derived hMSCs, with a final cell yield of 10^9^ cells and recovery rate of ~99%, upon microcarrier dissolution [35]. The stem cell immunophenotypic features, such as trilineage differentiation abilities and genome stability, were all preserved. Lastly, dissolvable gelatin-based microcarriers, have been recently developed by Xien Ng et al., who successfully grew MSCs in stirred-tank bioreactors at the three-liter scale, with significantly improved harvesting efficiency and speed, compared to conventional microcarriers [48]. Like previous reports, the multipotency of MSCs was retained post-harvesting. Since gelatin is a safe material for human contact, the authors suggested that rapid and safe cell release from the microcarriers would be feasible in larger-scale cell therapy manufacturing settings. However, the challenge is obtaining a recombinant source of gelatin, as current sources come from animal derived materials.

## 4. Biodegradable Microcarriers for Tissue Engineering Applications

Scaffolds are a stable framework, that are made of polymeric biomaterials, which enable cells to bind onto the scaffold, to secrete ECM proteins that imitate the support of structures (the biophysical and biochemical indices of indigenous tissue in which cells can grow), to migrate, and eventually to transform into tissues [39,40]. Substantial progress, and the development of advanced-engineered scaffold platforms, is needed for tissue repair applications, but growing large quantities of cells, ranging from millions to billions, with clinically amenable quality for therapies, remains a challenge to achieve [41,43]. Due to a paucity of cells in cell banks for clinical infusion, an effective platform for the biomanufacturing of cellular products is needed, to meet clinical demand [42,44].

Traditional tissue engineering approaches typically integrate three-dimensional (3D) scaffolds with cell sources and growth factors, to generate in vitro tissues. However, such tissue constructs have a history of failing to fill and heal irregularly shaped defects, such as cartilage replacement, thus restricting the clinical significance of tissue engineered products [45]. To address such technical constraints, engineered microtissues, with cell-laden microcarriers, have been designed to precisely match defect areas, as building blocks for implantable/injectable treatment. After implantation, microcarriers embedded in tailored microtissues provide a critical frame for establishing functional tissue growth and anastomosis (i.e., connection between adjacent tissue structures). In a recent study, dialdehyde bacterial cellulose (DBC), a natural material with nanofibrous characteristics, was used to develop ECM-mimicking microcarriers, which could simulate the matrix complexity of collagen, hydroxylysine, and chitosan. Thus, replicating cartilage ECM, and potentially enhancing tissue repair and regeneration. The effects of several parameters, on the nanofibrous microcarriers, such as chitosan concentration, porosity, as well as biomechanical profile and degradation properties, have also been evaluated. The cytocompatibility was confirmed in vitro, by examining cell proliferation and viability. Furthermore, these microcarriers were successfully used to create functional microtissues under microgravity culture conditions, and the cultured microtissues were applied in implantation experiments in Sprague–Dawley rats with a knee articular cartilage defect, in which effective cell proliferation, differentiation, and tissue recovery for cartilage repair was shown by implanted nanofibrous microcarriers, thus assessing the potential of microcarriers for cartilage regeneration [49].

The lack of biocompatible materials has hindered the advancement of biodegradable implants for bone tissue engineering. As a result, strengthening bioactivity through surface modification of the composite is critical for bone regeneration. BMP-2, a key component in initiating osteogenesis and facilitating bone repair, has been used extensively in clinical trials. Previous studies have found that the greater biodegradability of PLGA/HA nanocomposites, gives them higher biocompatibility and osteoconductivity properties for bone grafts. However, due to the polymers’ weak hydrophilicity and absence of functional groups, the growth factor loading efficiency is frequently reduced. Attempts were made to immobilize BMP-2 on graphene oxide (GO)-incorporated PLGA/HA (GO-PLGA/HA) biodegradable microcarriers. These biodegradable microcarriers also have the advantage of offering a substantial percentage of anchoring sites, which promotes cell adhesion. Chuan Fu et al. reported graphene oxide (GO)-promoted immobilization of peptides on PLGA/HA microcarriers, in less than 120 min; the cytocompatibility of MC3T3-E1 cells (murine cell line) cultivated on these microcarriers, resulted in significantly better cell adhesion and proliferation, via GO and HA [46]. Furthermore, the π-electron clouds of GO are capable of interacting with the inner hydrophobic cores of BMP-2 protein, improving the protein adsorption capacity and efficiently increasing BMP-2 binding on the microcarrier surface, allowing microcarriers to perform long-term osteoconductivity. Immobilization of BMP-2 on GO-PLGA/HA microcarriers, enhanced osteogenic differentiation to a greater extent, which was confirmed by alkaline phosphate activity, qRT-PCR, immunofluorescence staining, and mineralization on the deposited substrates. GO-PLGA/HA microcarriers delivered sustained BMP-2 activity, contributing to an improved osteogenic profile. Chitosan, conjugated with a lactose derivative containing non-toxic β-galactose moieties, increased chondrocyte aggregation, while also stimulating the creation of chondro-specific extracellular matrix (ECM) [50]. As a result, the microcarriers integrated with the chitosan-grafted lactose molecules were able to stimulate chondrogenesis, resulting in improved biological performance for cartilage repair.

Bioprinting is the process of printing scaffolds with embedded cells, to fabricate tissue constructs for regenerative medicine applications. Although bioinks with cells improve biomimetic features, issues still exist with ECM formation, cell activity, proliferation, and the ability to change into functional tissue constructs that resemble native tissue [51,52]. Although, bioprinting microcarriers seems a simple process, challenges, such as nozzle blockage, can arise when printing high cell densities. Microcarriers enable cells to self-assemble to high cell density within bioinks, and thus represent a favorable milieu for enhanced cell interaction, and fabrication of stable tissue constructs with more functional properties [53]. Bioinks with porous biodegradable microcarriers embedded within hydrogels, have generated functional osteochondral tissue structures with high cell density, at 8 × 10^6^/mL [54]. Levato R. et al., reported 3D printing of MSCs in PLA microcarriers, which exhibited significantly greater inter-cellular interaction and differentiation potential compared to hydrogels with only cells, and no microcarrier controls [53]. PLA microcarriers were pre-seeded with gelatin-methylacrylate and gellan gum (GelMA-GG) solution in one condition, whereas other PLA microcarriers were embedded with MSCs in GelMA-GG hydrogels for bioprinting. Cell viabilities of more than 90% have been reported after 3 days of culturing. Surprisingly, MSCs suspended with microcarriers in GelMA-GG hydrogels, attached to the surface of the microcarriers without the need for a pre-seeding step. The MSCs were observed to be at an early stage of adhesion onto the microcarriers after 4 h, in the presence of GelMA, whereas if seeded directly onto the microcarriers, they already expressed structured actin fibers [53]. Thus, cell-laden biodegradable microcarriers for bioprinting and tissue engineering, could serve as essential modular components for 3D printing functional tissue structures.

**Figure 2:**
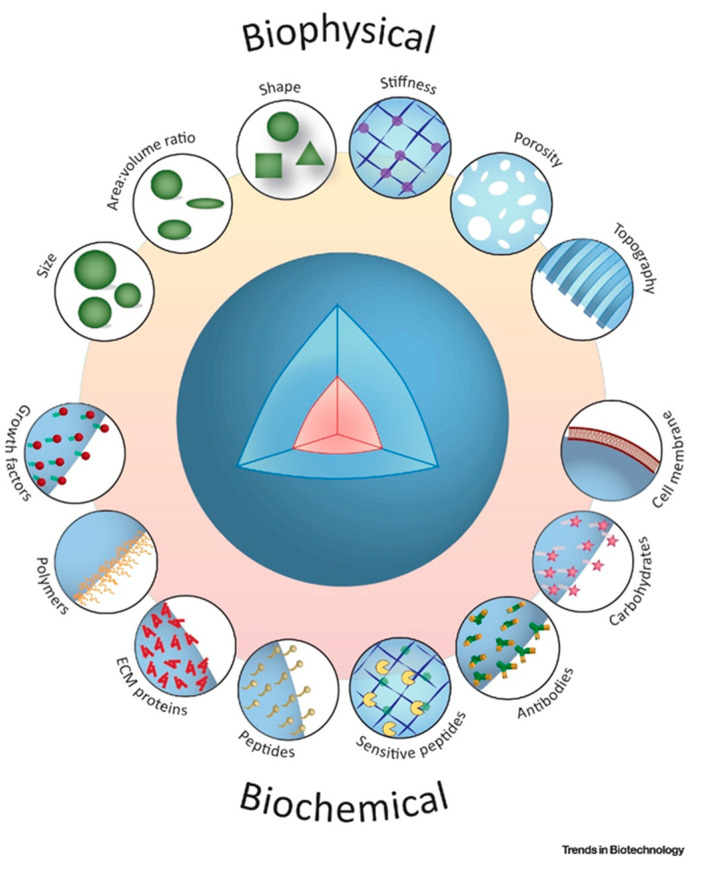
Schematic illustrations of biochemical and bio-physical indications contribute to the modulate topographical and architectural characteristics of biodegradable microcarriers for therapeutic applications. Adapted with permission from [58].

In the pursuit for a reliable and compliant cell expansion strategy, microcarriers with diverse physicochemical features have been designed. The shape and topographical features of microcarriers, such as interconnected pores, provide an expansive tissue-like microenvironment, that significantly improves cell growth and differentiation profiles [55] as illustrated in Figure 2. Engineered microcarriers can be configured to promote cell attachment and differentiation, and to be degradable at a controlled rate [56]. Optimization across a wide range of cell densities is needed, to achieve implantable microcarrier populations for injection. Although their handling can be described as straight-forward, hydrogel-only injectable systems often have poor mechanical stability and are not sufficiently durable to support proliferation and differentiation of anchorage-dependent cells, before formation of new tissue [57].

## 5. Biodegradable Microcarriers for Drug Delivery

Microcarriers have sparked a surge of interest in drug delivery, as the production of functional carriers utilizes simple procedures with new, but accessible, materials. The development of smart, bioactive and biodegradable microcarriers, is important for enhancing drug delivery and promoting tissue repair, and personalized medicine as a clinical norm [59,60]. Han Zhang et al.*,* reported novel soybean protein microcarriers, using a microfluidic strategy for drug delivery, the technology was inspired by the tofu production mechanism, of combining soymilk and brine for cross-linking soybean proteins. Since soybean protein droplets are synthesized via a microfluidic emulsification method, tofu microcarriers are relatively monodispersed and have homogeneous morphologies [61]. The impact of heating temperatures ranging from 20 °C to 90 °C, and brine concentrations ranging from 0.1% to 10%, on the optimal conditions for producing tofu, were explored. When the brine concentration was around 6%, the tofu had excellent morphologies, however, the texture of the tofu became tougher as the heating temperature increased. As a result, in subsequent studies, 6% brine and an 80 °C heating temperature was used.

Therapeutic cells can be delivered as living drugs by microcarriers, ideally in a spatiotemporally controlled manner. The ability to control the release of cells is important, because direct cell injection has been shown to result in greatly increased cell mortality, rendering the treatment ineffective [62,63]. Another promising application of injectable cell-laden microcarriers, is their use in the development of tissue models for targeted drug delivery research [64,65]. The use of advanced methods for delivering cells, to maximize the tissue repair potential, as well as to regenerate by stimulating angiogenic factors, has been demonstrated. Chara Simitzi et al. reported different surface topographies of hierarchically structured, porous biodegradable PLGA microcarriers, used for growing AdMSCs, and influence of microcarriers towards secretion of proangiogenic factors. Three different PLGA-based polymers, were used to fabricate microcarriers via thermally induced phase separation (TIPS) [57]. Briefly, AdMSCs were grown on all three compositions of PLGA-TIPS microcarriers, under xeno-free conditions, for 11 days, LDH assay confirmed the cell viability of around 95%, and the results were compared with cells grown on tissue culture (TC) plates. The ability of trilineage differentiation has also been demonstrated for cells grown on PLGA-TIPS microcarriers. Multiple proangiogenic factors, including VEGF, were also amplified in the secretome of AdMSCs grown on microcarriers, indicating their ability to trigger angiogenesis. By day 7 of the culture period, VEGF values (~5000 pg/mL) were almost 2–3 fold higher in PLGA-TIPS microcarriers, compared to the TC control. The functional properties of hierarchically organized, porous biodegradable microcarriers have been found to elevate the angiogenic potency of AdMSCs, to induce vascularization events, such as tubule formation and formation of branch points. Thus, PLGA-TIPS biodegradable porous microcarriers promote the secretion of proangiogenic factors towards inducing angiogenesis, offering a promising tool for neovascularization in ischemic tissue, when delivered in vivo.

**Figure 3:**
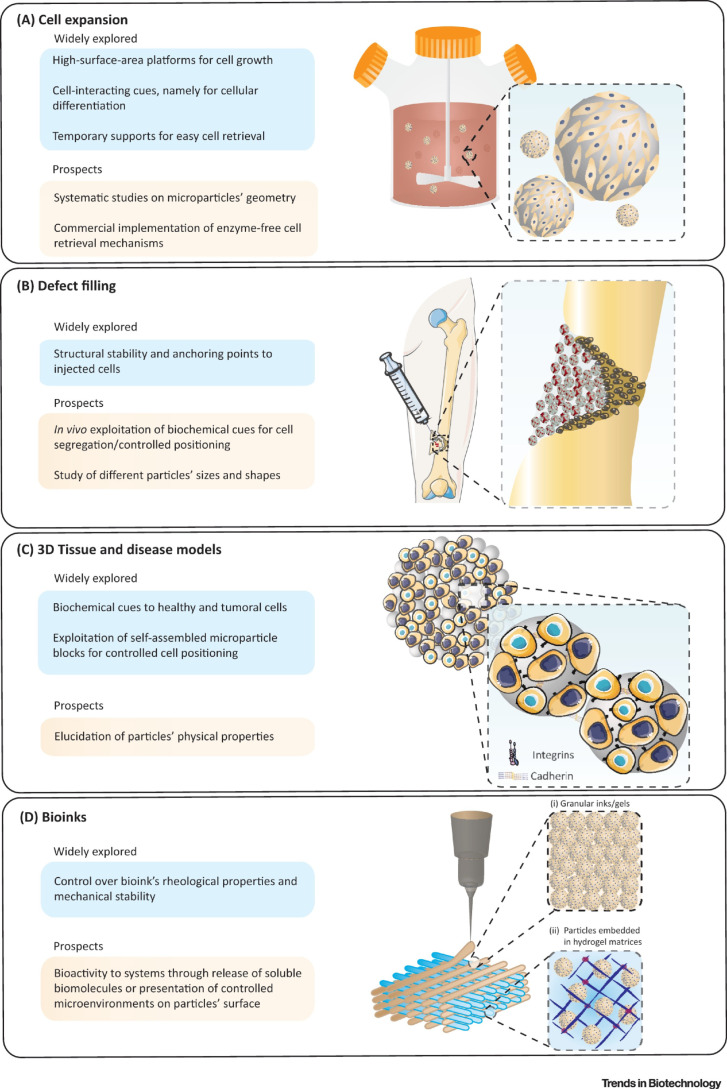
Schematic illustration to highlight: (**A**) use of microcarrier in bioreactors for mass expansion and differentiation of cells. (**B**) The microcarriers can be modified and injected into irregularly shaped defects, to effectively repair and enhance tissue recovery. (**C**) Microcarriers in multicellular aggregates, as structural supports, to promote cell growth and differentiation in the 3D system. (**D**) Advanced modular bioinks, that can accommodate (i) microcarriers tightly packed in the form of printable granular inks/gels; and (ii) microcarriers enabling the surrounding hydrogel matrix to mimic in vivo-like tissue architecture. Adapted with permission from [58].

As per the Figure 3 illustrations taken together, injectable biomaterials are promising candidates for fabricating a new class of biodegradable and injectable microcarriers that can generate and guide specific drug/cell responses, that match the biological environment towards defect filling, tissue repair and regeneration using 3D culture and bioprinting platforms [66,67].

## 6. Future Scope and Challenges

Therapeutic cells, potentially offer long-term cures for diseases and disorders that are not currently curable by conventional drugs and biological molecules [68,69]. This change in paradigm in modern medicine, can be achieved only if appropriate clinical-grade techniques can be developed for the large, cost-effective, and reproducible manufacturing of high-quality cells. A range of different natural and synthetic polymeric microcarriers have already been used for cell manufacturing. Most current research focuses on cell attachment and expansion [70]. Despite this potential, the sector is being held back by the range of challenges around the large-scale harvesting of cells from microcarriers, non-degradable materials used for microcarrier fabrication, as well as large-scale (kilogram level) manufacturability of microcarriers. Microcarriers must also be suitable for dehydration for dry storage, reconstitution in buffer, sterilization by autoclaving, and have a long shelf life (Figure 1).

Synthetic and natural biodegradable polymers such as the β-galactose moieties, oligosaccharides, sugars, and peptides are being considered for cell expansion, as well as effective cell recovery from microcarriers. Additionally, microcarriers are being explored to enhance tissue repair and regeneration [71].

Alternatively, nature inspired biodegradable materials such as coral reefs [50], novel chitosan–cellulose nanofiber [72], and plant inspired lignin-based cell adhesive hydrogels [73], are also being explored as microcarrier materials for cell manufacturing. Biodegradable microcarriers require fewer chemical reactants that need to be eliminated after implantation for tissue repair in a clinical setting, making them less expensive and ideal for in vivo application. In some cases of tissue repair and regeneration applications, cells must be seeded/embedded in a substrate that can provide a temporary matrix, to boost tissue regeneration [74,75]. Likewise, bio-inspired silk- and sericin-based microcarriers, in conjugation with bio-additives such as cellulose, dextran, pullulan, and many others which satisfy manufacturability, and bioactive and non-immunogenic properties for cell manufacturing applications, are being studied. In addition to natural polymers, inorganic complexes, such as calcium phosphates, have been used as well, to fabricate silk-based microcarriers [76]. Therefore, biodegradable microcarriers offer a broad and versatile platform for stem cell expansion, tissue regeneration, and drug delivery.

**Table 1:** Biodegradable materials used for microcarrier fabrication and applied for stem cell manufacturing, tissue engineering, and drug delivery applications.

Biodegradable Materials	Fabrication Technique	Microcarrier Characteristics	Cell Type and Use Case	Reference
**Cell expansion for therapy**				
**PLGA** + porcine gelatin coating	Emulsification of gelatin in PLGA/dichloromethane solution, followed by an emulsion-solvent evaporation method (25)	Porous 360 cm^2^/g d_50_ = 166 µm	Human adipose stem cells	[34]
**PLGA** + bovine gelatin or PLL coating	Single-emulsion solvent evaporation method followed by lyophilization	Porous d = 165–260 µm	Human umbilical vein endothelial cells	[74]
**Gelatin**	Droplet microfluidics (gelatin solution + fluorocarbon oil), followed by solidification in ice box	Non-porous d = 55–180 µm	Human mesenchymal stem cells	[48]
	Cross-linking commercial gelatin beads (CultiSpher G) using 1,2-bis(2-isocyanatoethyl) disulfide	Porous (pore size: 5–15 µm) d = 130–380 µm (hydrated)	Human mesenchymal stem cells	[72]
**PEG/alginate** + chitosan coating	Emulsification of sodium alginate and PEG (water phase) and Tween80/peanut oil (oil phase) peanut oil (oil phase)	Porous (pore size: 20–200 µm) d = 700–1900 µm	Human umbilical cord blood mesenchymal stem cells	[38]
**Poly-e-caprolactone (PCL)** + poly-l-lysine or fibronectin coating	Droplet microfluidics	Non-porous d = 150–170 µm	Stem cell expansion (WJ-MSC, hESCs) Tissue engineering (in vivo osteogenic differentiation)	[30,77,78,79]
**Chitosan**	Micro-emulsification of chitosan solution in oil phase followed by low-temperature thermally induced phase separation technique	Porous (pore size: 20–50 µm) d = 150 µm	Cell expansion (human fetal hepatocytes)	[80,81]
**Zein**	Zein ground in glycerin at 120 °C, 5 min, followed by the removal of glycerin by suction filtration. Finally, particles are repeatedly washed with pure water	Low porosity d = 150–230 µm 350 cm^2^/g 1.045 g/cm^3^	Cell expansion (vero cells)	[82]
**Cell expansion for tissue engineering**				
**PLGA/hydroxyapatite + incorporated graphene**	Emulsion-solvent evaporation, followed by surface immobilization of BMP-2	Non-porous d_50_ = 520 µm	Osteogenesis	[46,83]
**PLGA** + poly-l-lysine coating	Emulsion-solvent evaporation method, followed by surface immobilization of BMP-7 and ponericin G1	Non-porous d_50_ = 560 µm	Osteogenesis	[83]
**Poly-lactic acid (PLA)** + human recombinant collagen type I coating	Emulsion/solvent (ethyl-lactate) evaporation technique	Non-porous d = 82 ± 23 µm	Rat bone marrow MSCs	[32]
**Cellulose/chitosan** (cross-linked)	Water phase consisting of cellulose and chitosan solution emulsified into microspheres in liquid paraffin (oil phase) under stirring, followed by phase separation through liquid nitrogen quenching and petroleum ether	Porous (pore size: 30–60 µm) d = 450 um	Bone marrow derived MSCs for cartilage regeneration	[72]
**Drug delivery**				
**Tofu/soybean protein**	Capillary microfluidic (emulsification) device, followed by thorough ethyl alcohol wash to get rid of soybean oil from the bead’s surface	Porous d = 640–740 µm	Drug delivery	[61]
**Pectin**	Electrospraying pectin solution into solution of cross-linking mixture (CaCl2/oligochitosan), followed by rinsing with DI water	Porous d = 150–600 µm	Drug delivery	[47]

**Table 2:** Quantified data set highlighting the different surface coatings used on microcarriers for enhanced cell attachment, bead-to-bead transfer, and applications in cell differentiation or expansion.

Surface Coating	Type of Microcarrier	Surface Coverage (%)	Cell Attachment Efficiency (%)	Bead-to-Bead Transfer Efficiency (%)	Differentiation Potential	Reference
Polylysine	Chitosan	95	85	80	Adipose derived stem cells; nerve guide conduits	[84]
Poly(N-vinylguanidine)	Polystyrene	80	75	85	Expansion of human mesenchymal stem cells	[85]
Poly(N-isopropylacrylamide) (PNIPAAm)	Polycaprolactone (PCL)	70	80	90	Fibroblasts and mesenchymal stem cell expansion	[86]
Genipin	Chitosan/Alginate	60	70	75	Expansion of mesenchymal stem cells	[87]
PNIPAAm	Alginate	50	60	70	Expansion of umbilical cord derived mesenchymal stem cells	[38,88]
PLL	PLGA	90	80	90	Expansion of HUVEC cells and umbilical cord derived mesenchymal stem cells	[89,90]
Gelatin	Polystyrene	95	90	95	Expansion of mesenchymal stem cells	[37]
PEG	PLGA	80	75	80	Expansion of mesenchymal stem cells	[91]
Chitosan	Alginate	60	70	80	Expansion of L929 and Mesenchymal stem cells	[92]

## Data Availability

Not applicable.
